# Personalized Drug Repurposing Screen Identifies Patient-Specific Therapeutic Candidates for Mucopolysaccharidosis Type IIIB

**DOI:** 10.3390/jpm16070369

**Published:** 2026-07-08

**Authors:** Kathleen D. McDaniel, Neda Ghousifam, Rodney A. Bowling, Catherine Z. Chen, Wei Zheng, Zeenat A. Shyr

**Affiliations:** 1 National Center for Advancing Translational Sciences, National Institutes of Health, 9800 Medical Center Drive, Rockville, MD 20850, USA; 2 AlphaRose RareLabs, 1401 Lavaca St, Austin, TX 78701, USA

**Keywords:** mucopolysaccharidosis type IIIB, Sanfilippo syndrome, personalized medicine, drug repurposing, high-content screening, lysosomal storage disease, rare disease, patient-derived fibroblasts, precision medicine, genetic heterogeneity

## Abstract

**Background:** Mucopolysaccharidosis type IIIB (MPSIIIB, Sanfilippo syndrome type B) is a rare lysosomal storage disease caused by deficiency of alpha-N-acetylglucosaminidase (NAGLU) enzyme, leading to progressive accumulation of heparan sulfate and severe neurological decline. MPSIIIB’s significant genetic heterogeneity presents a major barrier to developing broadly effective treatments and suggests a need for personalized therapeutic strategies. **Methods:** We established a personalized drug repurposing platform using high-content imaging with lysotracker dye as an indirect functional readout of lysosomal dysfunction to screen compounds that correct lysosomal defects in patient-derived fibroblasts. We screened 2807 compounds on cells from an MPSIIIB patient with a homozygous NAGLU p.Arg297Ter mutation. Hits that reduced lysosomal accumulation by at least 25% with minimal cytotoxicity were validated and subsequently tested for efficacy in fibroblasts from a second patient with a different NAGLU genotype. **Results:** The primary screen yielded 72 hits (2.6% hit rate), with 10 confirmed in dose–response assays. Notably, four clinically approved drugs—baclofen, dextrose, epalrestat and moxifloxacin—reduced lysosomal accumulation in the index patient’s cells. However, none of these four drugs were effective in the second patient’s cells, demonstrating a profound patient-specific effect. Only one non-clinical compound, 6-chlorothymol, showed a trend toward activity in both cell lines. **Conclusions:** Our study demonstrates a feasible framework for conducting rapid, N-of-1 drug repurposing screens for rare diseases. While we identified four promising candidates for the index patient, the lack of efficacy in a second patient cell line underscores that genetic heterogeneity may preclude a “one-size-fits-all” approach for MPSIIIB. These findings support the integration of individualized drug screening as a potential precision-medicine strategy, offering a potential path toward patient-specific therapies for rare diseases rather than traditional drug development.

## 1. Introduction

Mucopolysaccharidosis type IIIB (MPSIIIB), also known as Sanfilippo syndrome type B, is a rare and devastating lysosomal storage disease caused by a deficiency of the alpha-N-acetylglucosaminidase (NAGLU), an enzyme required for the stepwise lysosomal degradation of glycosaminoglycan heparan sulfate (HS). HS proteoglycans at the cell surface and extracellular matrix undergo continuous turnover; following extracellular remodeling, proteolytic processing, or endocytic uptake, their HS chains are delivered to the endolysosomal system, where they are degraded sequentially by lysosomal enzymes [1,2]. In MPSIIIB, NAGLU deficiency blocks this degradative pathway, resulting in the progressive lysosomal accumulation of partially degraded HS, disruption of cellular homeostasis, and a severe neurological phenotype [3]. While clinically heterogeneous, MPS IIIB is primarily characterized by profound neurodegeneration, hyperactivity, sleep disturbances, and a decline in cognitive and motor function [4].

Currently, no approved therapeutics exist for MPSIIIB, and patient care is limited to symptomatic management. The primary therapeutic strategies explored for LSDs are enzyme replacement therapy (ERT) with recombinant enzyme, substrate reduction therapy (SRT) to inhibit the enzyme that catalyzes the biosynthesis of the burden metabolite, and pharmacological chaperone therapy to stabilize and improve the activity of the disease-causing protein (PCT) [5]. While ERT with recombinant NAGLU is a logical approach, it is hampered by the need for direct, invasive intracerebroventricular (ICV) administration to bypass the blood–brain barrier (BBB) and a high burden of treatment and cost. Although an ICV-delivered ERT showed promise in preclinical models [6] and in phase I/II clinical trials [7], its development was not advanced to gain FDA approval due to business decisions. AAV-based gene therapy, another promising modality, has also faced setbacks in clinical trials, which were terminated for reasons other than safety concerns [8]. The collective failure of these “one-size-fits-all” approaches suggests a more fundamental obstacle may be at play.

With over 300 mutations, including missense, nonsense, deletions, insertions, and splice-site mutations, identified in the *NAGLU* gene, the allele frequencies of the different mutations are quite low, possibly contributing to the spectrum of clinical symptoms [9]. This level of complexity is analogous to diseases such as cancer, where it is well-established that cellular and genetic heterogeneity necessitates personalized treatment strategies. The downstream cellular consequences of lysosomal storage are multifaceted, impacting autophagy, mitochondrial function, and inflammatory signaling pathways that may differ between patients [10]. It is therefore reasonable to hypothesize that therapeutic responses will be similarly individualized.

Here, we pivot from a universal to a personalized therapeutic approach. Our goal was to establish a rapid and scalable drug-repurposing platform using patient-derived cells. We leveraged patient fibroblasts, a well-characterized model that exhibits deficiency of the NAGLU enzyme [11], to study lysosomal storage defects and to screen for compounds capable of correcting the cellular phenotype. LysoTracker dye was selected for the primary screening assay. Under live-cell conditions, it accumulates in acidic organelles and serves as an indirect functional readout of lysosomal expansion and morphological changes caused by HS accumulation. This approach is well-established in published *in vitro* MPSIII disease models [12] and provides a robust, quantifiable signal window between MPSIIIB and healthy cells that is amenable to high-throughput workflows. We conducted a drug repurposing screen using the NCATS Pharmaceutical Collection on fibroblasts from an MPSIIIB patient. We identified several compounds that reduced lysosomal accumulation in the index patient’s cells without causing cytotoxicity. Notably, however, these hits did not produce the same effect in fibroblasts from a second MPSIIIB patient with a different NAGLU genotype. These findings not only demonstrate the feasibility of N-of-1 drug repurposing screens but also provide compelling evidence that a truly personalized approach may be essential for meaningful therapeutic progress in rare diseases like MPSIIIB.

## 2. Materials and Methods

### 2.1. Patient Information

Deidentified patient-derived fibroblasts were obtained through collaboration with AlphaRose RareLabs (Austin, TX, USA), a nonprofit organization focused on personalized medicine development for rare disease patients. The index patient fibroblast line was derived from an MPSIIIB patient harboring a homozygous c.889C>T (p.Arg297Ter) pathogenic variant in both NAGLU alleles, a nonsense mutation that abolishes NAGLU enzyme function. An age- and sex-matched healthy control fibroblast line (normal human dermal fibroblasts, NHDF) was obtained from the same source. A second MPSIIIB patient fibroblast line, GM01426, was purchased from the Coriell Institute for Medical Research (Camden, NJ, USA). The GM01426 fibroblast line has reduced α-N-acetylglucosaminidase activity and is homozygous for the NAGLU c.457G>A variant, resulting in the p.Glu153Lys (E153K) substitution [13]. This variant has been reported in ClinVar as likely pathogenic or pathogenic (Variation ID: 1067431; VCV001067431.11).

### 2.2. Cell Culture

Fibroblasts were cultured in DMEM supplemented with GlutaMAX™ and pyruvate (Thermo Fisher Scientific, Waltham, MA, USA, Cat# 10569010), 10% HyClone Characterized Fetal Bovine Serum (FBS; Cytiva, Marlborough, MA, USA, Cat# SH30071.03), and 1% penicillin-streptomycin (Gibco, Waltham, MA, USA, Cat# 15140-122). Cells were maintained in tissue culture flasks at 37 °C in a humidified incubator with 5% CO_2_ and 5% O_2_. Growth medium was replaced every 3 days, and cells were passaged at approximately 75–80% confluence.

### 2.3. Lysotracker Staining

All liquid handling steps were performed using angled dispense tips to prevent cell detachment. Following 72-h compound incubation, 50 nM LysoTracker Red DND-99 (Thermo Fisher Scientific, Cat# L7528) was added directly to live cells in complete growth medium and incubated for 1 h at 37 °C with 5% CO_2_ and 5% O_2_. This live-cell staining step ensures that LysoTracker accumulates in acidic organelles under physiological conditions. Cells were subsequently fixed with 4% PFA for 20 min at room temperature to stabilize the LysoTracker signal for high-content imaging; this sequential live-stain/post-fixation approach is standard practice in high-content screening for lysosomal storage diseases and does not affect the LysoTracker signal acquired under live-cell conditions. No surface coating (e.g., poly-D-lysine) was applied to the 384-well plates, and standard tissue-culture-treated surfaces provided adequate cell adherence and phenotypic stability at this format. Plates were washed four times with 70 µL per well of Dulbecco’s phosphate-buffered saline (DPBS) using a BioTek EL406 plate washer (Agilent Technologies, Santa Clara, CA, USA). Following washes, cells were counterstained with 1 µg/mL HCS CellMask Green (Thermo Fisher Scientific)—a dye used for cell boundary segmentation in the image analysis pipeline—and 4 µM Hoechst 33342 (Thermo Fisher Scientific)—a nuclear dye used for nuclei identification—in DPBS for 30 min at room temperature. Plates were washed twice with 70 µL per well of DPBS, sealed, and imaged immediately. Images were acquired from nine fields per well using the Opera Phenix High-Content Screening System (Revvity, Waltham, MA, USA) with a 20× or 40× water-immersion objective.

### 2.4. Nile Red and Heparan Sulfate Staining

Nile Red staining was performed similarly to lysotracker staining with the following modifications: 1.5 µM Nile Red dye (Thermo Fisher Scientific) was added to live cells in place of LysoTracker, and 4 µM Hoechst 33342 (Thermo Fisher Scientific) was added together with 4% PFA during fixation. Following post-fixation washes, plates were sealed and imaged as described above. For heparan sulfate staining, cells were fixed in 4% PFA, then permeabilized with 0.1% Triton-X (Sigma, St. Louis, MO, USA) for 10 min and blocked in Cell Staining Buffer (Biolegend, San Diego, CA, USA) for one hour at room temperature. Cells were incubated with Heparan sulfate antibody (Cat# F58-10E4, Amsbio, Cambridge, United Kingdom) overnight at 4 °C at 1:50 dilution. After overnight incubation, cells were washed with PBS before incubating with Alexa-488 secondary antibody (1:1000; Thermo Fisher Scientific) for one hour at room temperature. Cells were rinsed with PBS and incubated with 4 μM Hoechst 33342 (Thermo Fisher Scientific) and CellMask red (Thermo Fisher Scientific) for 30 min at room temperature. After washing with PBS, plates were sealed and imaged with the Opera Phenix Plus High-Content Screening System using a 40× water objective (Revvity, Waltham, MA, USA).

### 2.5. Whole Well Fluorescence Measurement

Cells were seeded into 384-well black solid-bottom plates at the optimized density in 30 µL of growth medium per well using a Multidrop Combi Liquid Dispenser (Thermo Fisher Scientific). Following 72-h incubation, 625 nM LysoTracker Red DND-99 (Thermo Fisher Scientific, Cat# L7528) was added directly to the growth medium and cells were incubated for 1 h at 37 °C with 5% CO_2_ and 5% O_2_. Fluorescence intensity was measured using a CLARIOstar plate reader (BMG Labtech, Cary, NC, USA).

### 2.6. Compound Library

The NCATS Pharmaceutical Collection (NPC) is an in-house library of 2807 small-molecule compounds that are registered or approved for use in humans, animals, or both. The library comprises 49% U.S. FDA-approved drugs; 23% drugs approved in the EU, Canada, or Japan; and 28% compounds in clinical trials or used as research tools [14].

### 2.7. Primary and Confirmation Screening

Cells were seeded in black, clear-bottom PhenoPlate™ 384-well microplates (Revvity, Cat# 6057302, Waltham, MA, USA) at a density of 600 cells per well in 10 µL of growth medium using a Multidrop Combi Liquid Dispenser (Thermo Fisher Scientific). Plates were incubated overnight (18–20 h) at 37 °C with 5% CO_2_ and 5% O_2_ prior to compound addition. Compounds were transferred from source plates using an Echo Acoustic Liquid Handler (Beckman Coulter, Inc., Brea, CA, USA), dispensing 30 nL per well. NHDF control cells were plated in column 1 on every assay plate and served as an internal reference for basal LysoTracker intensity. NHDF were not subjected to further screens. MPSIIIB patient cells were plated in columns 2–24. Dimethyl sulfoxide (DMSO) vehicle controls were dispensed in columns 1 and 2. DMSO vehicle controls produced no significant change in LysoTracker staining intensity or cell viability relative to untreated wells, confirming no solvent effect at the concentrations used. Following compound addition, 20 µL of growth medium was added per well using an angled-tip Multidrop Combi Liquid Dispenser. Plates were incubated for 72 h at 37 °C with 5% CO_2_ and 5% O_2_ before proceeding with the lysotracker staining protocol.

For the primary screen, each compound was tested at a single concentration of 10 µM in duplicate (two biological replicates). Compounds were dispensed in columns 3–24. For confirmation assays, selected hits were further evaluated for a dose response in four or more biological replicates. Data from compound-treated MPSIIIB wells were normalized to DMSO-treated MPSIIIB control wells and expressed as percent change from untreated controls.

### 2.8. Cell Viability Assays

Cell viability was assessed using CellTiter-Glo Luminescent Cell Viability Reagent (Promega, Madison, WI, USA). Cells were seeded into 384-well white solid-bottom plates at 600 cells per well in 10 µL of growth medium using a Multidrop Combi Liquid Dispenser (Thermo Fisher Scientific). After overnight incubation, cells were treated with compounds and 20 µL of growth medium was added. Following 72-h incubation, 30 µL of CellTiter-Glo reagent was added to each well. Plates were incubated for 10 min at room temperature, and luminescence was measured using a PHERAstar plate reader (BMG Labtech, Cary, NC, USA). Data were normalized to wells containing 0.5% DMSO (100% viability control) and wells containing growth medium only (0% viability control).

### 2.9. Image Analysis and Quantification

Images were acquired using the Opera Phenix High-Content Screening System (Revvity, Waltham, MA, USA; see Section 2.3) and transferred to Image Artist software, version 1.5.1 (Revvity, Inc.) for batch analysis. Nuclei were first identified based on Hoechst 33342 staining. This was followed by cytoplasmic segmentation using CellMask Green fluorescence (Alexa Fluor 488 channel) to define the exact cell boundaries. LysoTracker-positive puncta (Alexa Fluor 568 channel) within the cytoplasm were detected as spots within each cell. Spots were analyzed for number, intensity, and area individually. To resolve individual boundaries within lysosomal clumps using the automated software, we captured the overall multidimensional phenotypic complexity of lysosomal expansion in MPSIIIB cells (where increased lysosomal number, enlarged size, and heightened acidity all contribute simultaneously to the disease phenotype) by using a composite metric: (number of LysoTracker spots) × (LysoTracker spot area in µm^2^) × (LysoTracker mean spot intensity in the Alexa Fluor 568 channel). This measure is referred to as “integrated lysotracker intensity” in this study.

### 2.10. Statistical Analysis

All statistical analyses were performed using GraphPad Prism version 10.0.1 (GraphPad Software, San Diego, CA, USA). Data are presented as Mean ± Standard Error of the Mean (SEM). Statistical comparisons were performed using Student’s *t* test or one-way ANOVA with Tukey’s multiple comparisons test. Statistical significance was defined as *p* < 0.05 (*, *p* < 0.05; **, *p* < 0.01; ***, *p* < 0.001; ****, *p* < 0.0001).

## 3. Results

### 3.1. Development of a Robust High-Content Assay for Lysosomal Storage

To enable a personalized drug screen, we first developed a robust high-content imaging assay to quantify accumulation in patient-derived fibroblasts as an indirect functional readout of glycosaminoglycan (GAG)-driven lysosomal dysfunction. LysoTracker, a fluorescent dye that accumulates in acidic organelles, measures the downstream functional consequences of HS accumulation—including lysosomal expansion, morphological changes, and increased acidification—rather than HS levels directly. This assay has been established and validated in published in vitro MPSIII disease models [12] and was selected here following systematic comparison with alternative readouts, as described below. Critically, LysoTracker is added to live cells prior to fixation under fully physiological conditions and only subsequently fixed with 4% PFA to stabilize the signal for high-content imaging. This sequential live-stain/post-fixation protocol is standard in high-content screening for lysosomal storage diseases and ensures the signal reflects genuine physiological lysosomal acidification. Initial attempts to optimize the assay in a 1536-well format for maximum throughput were unsuccessful, as both MPSIIIB and healthy control (NHDF) fibroblasts exhibited poor adherence, cell loss, or altered morphology attributable to the poly-D-lysine (PDL) coating applied in that format (Figure S1).

**Figure 1:**
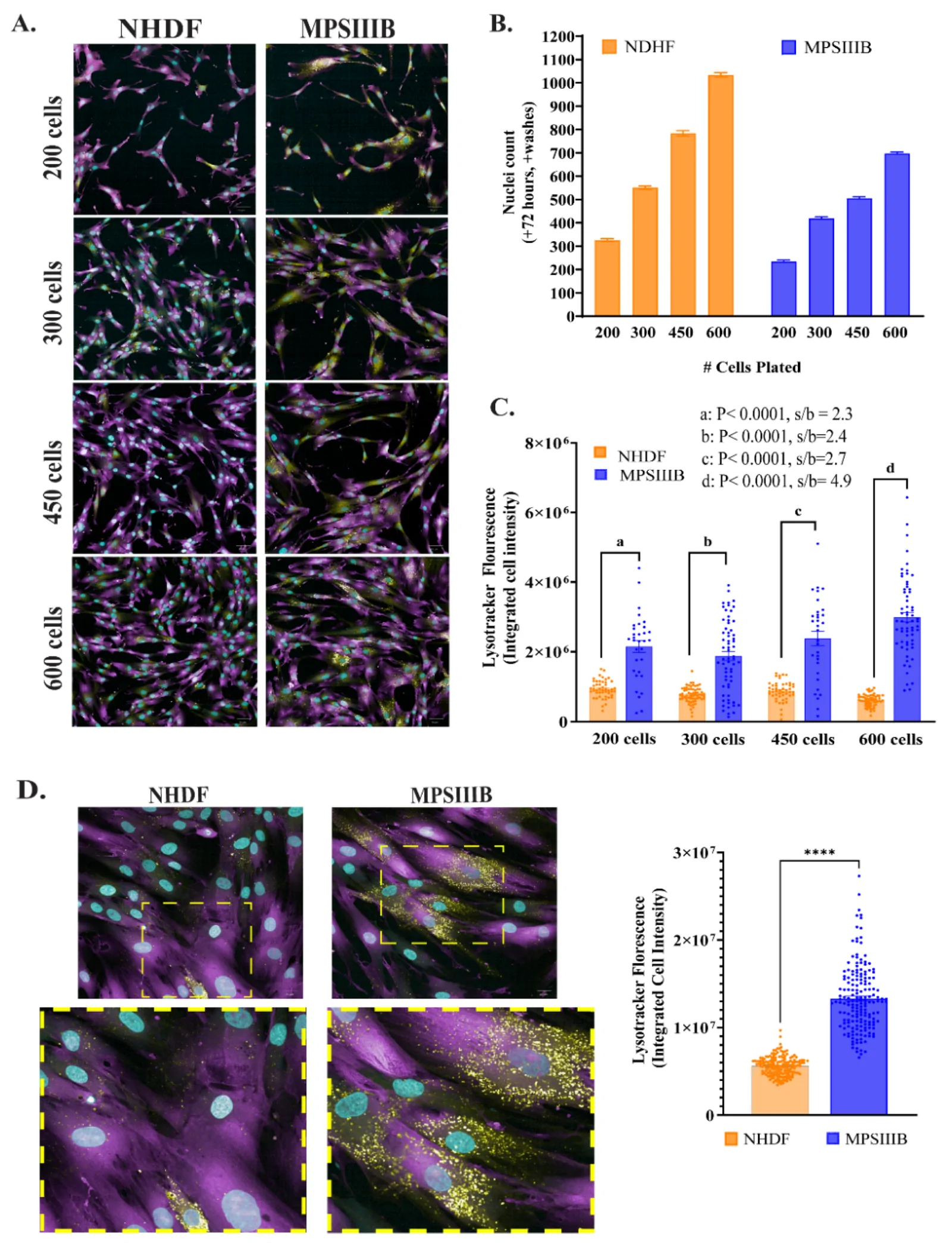
Assay optimization for cell density and imaging. (**A**) Representative images taken with a 20× water objective. Cyan, nuclei; magenta, high-content cell mask; yellow, LysoTracker. (**B**) Nuclei count for cell densities after 72 h averaged from 48 to 64 independent wells; nuclei count confirms no significant cell loss across densities in either cell line, serving as the viability readout for this experiment. (**C**) Quantification of integrated lysotracker intensity for 200, 300, 450 or 600 cells per well, which was calculated using LysoTracker spot number, intensity and area for each cell identified by nuclear staining; each data point represents an independent well averaged from 7 fields of view. *p* < 0.0001 by Student’s *t*-test for a, b, c and d comparisons between cell densities. (**D**) Images taken with 40× water objective. Top left: Representative image for 600 cells per well. Bottom left: magnified view of areas with lysotracker staining shown in yellow boxes. Right: Quantification for 600 cells per well; each data point represents an independent well averaged from 19 fields of view. **** *p* < 0.0001 by Student’s *t*-test. Data are presented as mean +/− SEM.

We therefore transitioned to a 384-well format, which markedly improved cell adherence and phenotypic stability without requiring any additional surface coating. We optimized cell density and imaging parameters, observing minimal cell loss and a stable phenotype. The three fluorescent channels shown in Figure 1A served distinct purposes: Hoechst 33342 (pseudo-color, cyan) was used to identify nuclei; HCS CellMask Green (pseudo-color, magenta) was then used to delineate cytoplasmic boundaries for cell segmentation; and LysoTracker Red (pseudo-color, yellow) marked lysosomal puncta within the cytoplasm.

Nuclei count served as a direct cell viability readout and confirmed that no significant cell loss occurred across tested densities in either NHDF or MPSIIIB cells, although the MPSIIIB exhibited slower growth in culture compared with NHDF (Figure 1B). As expected, fibroblasts from the MPSIIIB patient displayed a dramatic increase in lysosomal accumulation compared to healthy controls, characterized by a significant elevation in all three individual parameters—number, size, and fluorescence intensity of LysoTracker-positive puncta relative to NHDF controls (Figure S2). To accurately capture this complex, multidimensional phenotype, we developed an image analysis pipeline to calculate the Integrated LysoTracker Intensity—a composite metric of spot number, area, and mean intensity—for each cell (Figure 1C). Significant differences between NHDF and MPSIIIB cells (*p* < 0.0001) were evident at all cell densities tested: 200, 300, 450, and 600 cells. However, the signal-to-basal (s/b) ratio was best achieved with 600 cells per well, with a 4.9-fold difference (Figure 1C). Cell heterogeneity was evident in both NHDF and MPSIIIB cells, even when imaged at higher magnification using a 40× objective (Figure 1D). Nevertheless, the composite metric provided a robust and statistically significant window between diseased and healthy cells (Figure 1C,D). All subsequent assays were performed using 600 cells per well and 20× imaging, which enabled higher-throughput acquisition while preserving a robust disease-versus-control assay window.

**Figure 2:**
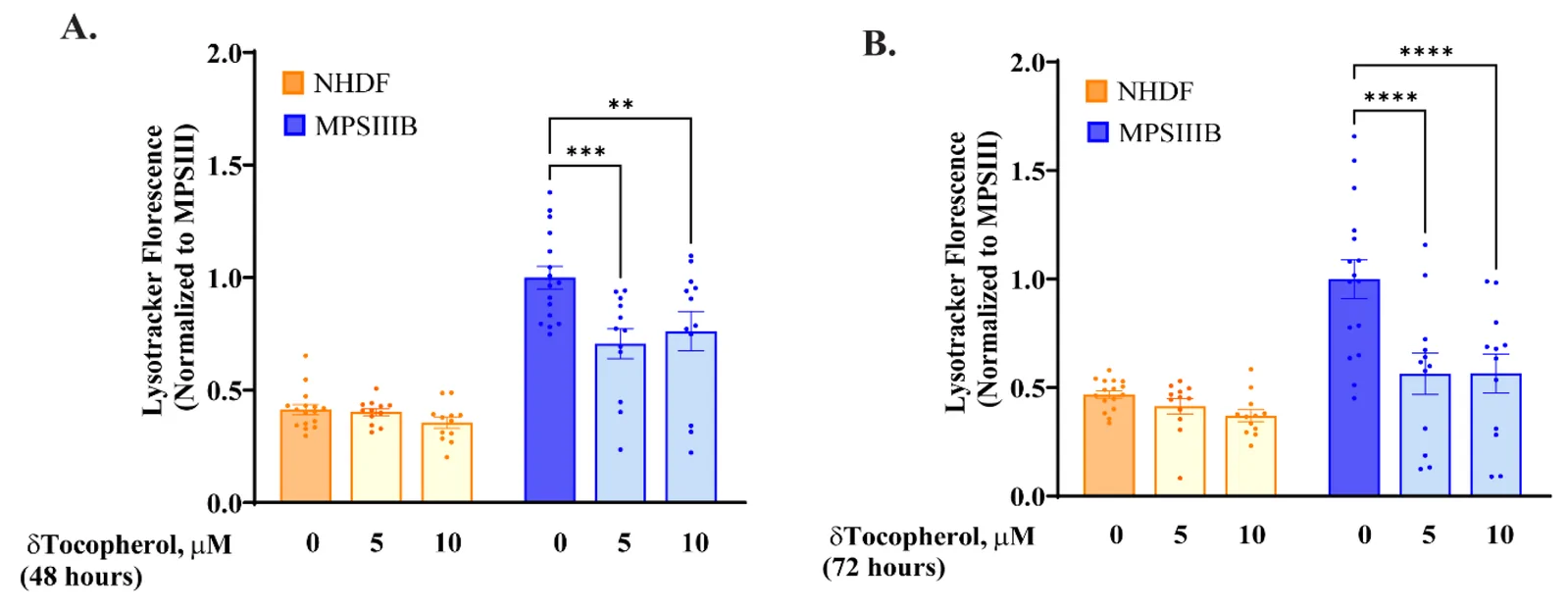
Assay optimization for drug incubation time. Integrated lysotracker intensity (**left**) and nuclei (**right**) after incubation with 5 μM or 10 μM δ-tocopherol for (**A**) 48 h and (**B**) 72 h. Each data point represents an independent well that was averaged from 7 fields of view. Data are presented as mean +/− SEM. ** *p* < 0.01, *** *p* < 0.001, **** *p* < 0.0001) by one-way ANOVA with Tukey’s multiple comparisons test.

To confirm the validity of this assay and to determine optimal drug incubation time, we treated cells with 5 µM and 10 µM δ-tocopherol, a compound previously shown to reduce lysosomal substrate accumulation in MPSII [15] and MPSIII [12] models. In the NHDF cells, treatment with 5 µM and 10 µM δ-tocopherol did not elicit any further reduction in LysoTracker staining after 48 or 72 h of treatment (Figure 2A,B). Both 48-h (Figure 2A) and 72-h (Figure 2B) incubation times with δ-tocopherol significantly reduced LysoTracker staining intensity in MPSIIIB cells compared to vehicle-treated MPSIIIB cells. The magnitude of reduction was greater after 72 h, with both 5 µM and 10 µM δ-tocopherol reducing staining to levels statistically indistinguishable from NHDF controls. These results are fully consistent with published live-cell LysoTracker data in MPS models [12,15], further validating the fidelity of the live-stain/post-fixation protocol. Based on these results, a 72-h drug incubation period was therefore selected for library screening in MPSIIIB cells.

To ensure we had not overlooked a more suitable assay method, we evaluated several alternative approaches for quantifying differences between NHDF and MPSIIIB cells. Previous *in vitro* studies of MPSIIIB have shown that neutral lipids, including cholesterol and glycolipids, can accumulate in lysosomes as a secondary consequence of heparan sulfate accumulation [12]. We tested whether Nile Red staining, which detects multiple lipid species, including cholesterol, neutral lipids, and glycolipids, might provide better assay performance. Although Nile Red staining showed significant differences between NHDF and MPSIIIB cells (Figure S3A), high variability persisted within the MPSIIIB population. Secondary substrate accumulation in MPS patient fibroblasts can vary substantially [16]. Likewise, secondary lipid burden can be heterogeneous and highly assay-dependent. These characteristics rendered Nile Red less suitable as a primary readout for high-throughput screening in our MPSIIIB cell model, though the data confirmed that secondary lipid accumulation was detectable in this model. We also evaluated whole-well LysoTracker fluorescence intensity using a plate reader (Figure S3B). Although MPSIIIB cells showed significantly higher fluorescence than controls, the signal-to-basal ratio was insufficient for robust screening. Given that NAGLU deficiency impairs lysosomal HS degradation in MPSIIIB, we assessed whether HS accumulation could be detected in patient-derived fibroblasts by immunofluorescence staining. Previous studies have reported HS accumulation within lysosomes, with abundant HS signal also detected at the cell surface and in the extracellular matrix of MPSIIIB cells [17,18]. Consistent with these reports, we observed pronounced, localized HS staining on the cell membrane and within the extracellular matrix of MPSIIIB fibroblasts (Figure S4). When quantifying intracellular HS fluorescence intensities, automated image quantification did not detect statistically significant differences between MPSIIIB and NHDF cells. The technical limitations of antibody-based HS detection, particularly via immunofluorescence, are well known. Reliable HS visualization can be compromised by several factors, including variable epitope accessibility resulting from differences in sulfation patterns and chain length, heterogeneous intracellular HS distribution, and the sensitivity of GAGs to fixation and multiple wash procedures associated with typical antibody-based immunofluorescence protocols. Additionally, immunofluorescence staining with HS antibody alone could not be reliably used for segmentation and intensity calculations for the cell membrane. It is important to note that automated image analysis algorithms have inherent limitations when applied to signals with irregular morphology, heterogeneous spatial distribution, or diffuse object boundaries. Such features may be readily discernible by visual inspection yet remain difficult to quantify accurately using pixel-based algorithms. This was evident for HS immunofluorescence in MPSIIIB fibroblasts, where qualitative differences were visible but were not robustly captured by automated analysis. Although conventional pixel-based image analysis was unable to reliably quantify these differences, advances in artificial intelligence (AI)-based image segmentation and feature extraction may improve the detection and quantification of such complex biological signals. Future studies leveraging these approaches may enable more sensitive and reproducible quantification of HS accumulation in high-content imaging applications.

In contrast, the LysoTracker assay produced a distinct signal consisting of discrete punctate lysosomal structures that were highly amenable to automated spot-detection algorithms. Consequently, this platform identified highly significant differences between MPSIIIB and NHDF cells across all cell densities tested (*p* < 0.0001; Figure 1C,D). Because a primary screening assay requires a signal that can be quantified reproducibly and at scale, the comparative assessment of multiple readouts supported LysoTracker-based high-content imaging in 384-well plates as the optimal approach. This assay provided the most robust and reproducible indirect measure of the disease-associated lysosomal phenotype in this cellular model.

### 3.2. Primary Screen Identifies Clinically Approved Drugs That Correct the Lysosomal Phenotype

**Figure 3:**
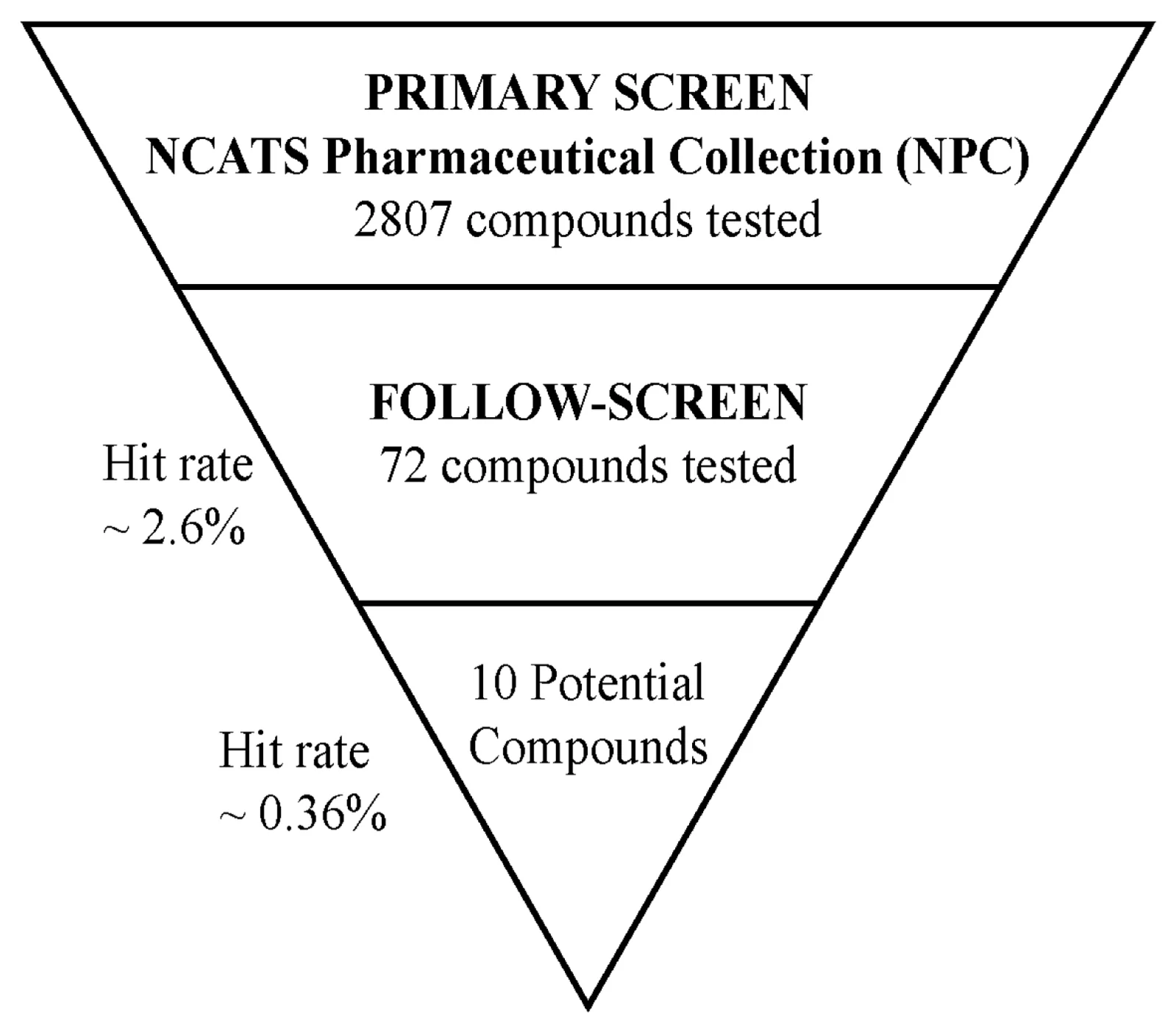
Drug screen outcome: Triangle plot for screening results with total compounds screened, number of primary hits and confirmed hits. The selection criteria were compounds showing an average of at least 25% reduction in lysotracker integrated staining intensity with less than 50% cell loss.

Using this optimized platform, we screened the 2807-compound NCATS Pharmaceutical Collection (NPC) at a single 10 µM concentration in replicates in MPSIIIB fibroblasts. The primary screen identified 72 compounds that reduced Integrated LysoTracker intensity—as an indirect measure of lysosomal dysfunction—by over 25% with less than 50% cytotoxicity, yielding a hit rate of 2.6% (Figure 3). These hits were re-tested in confirmation staining and viability assays, from which 10 compounds were validated (Table 1).

**Figure 4:**
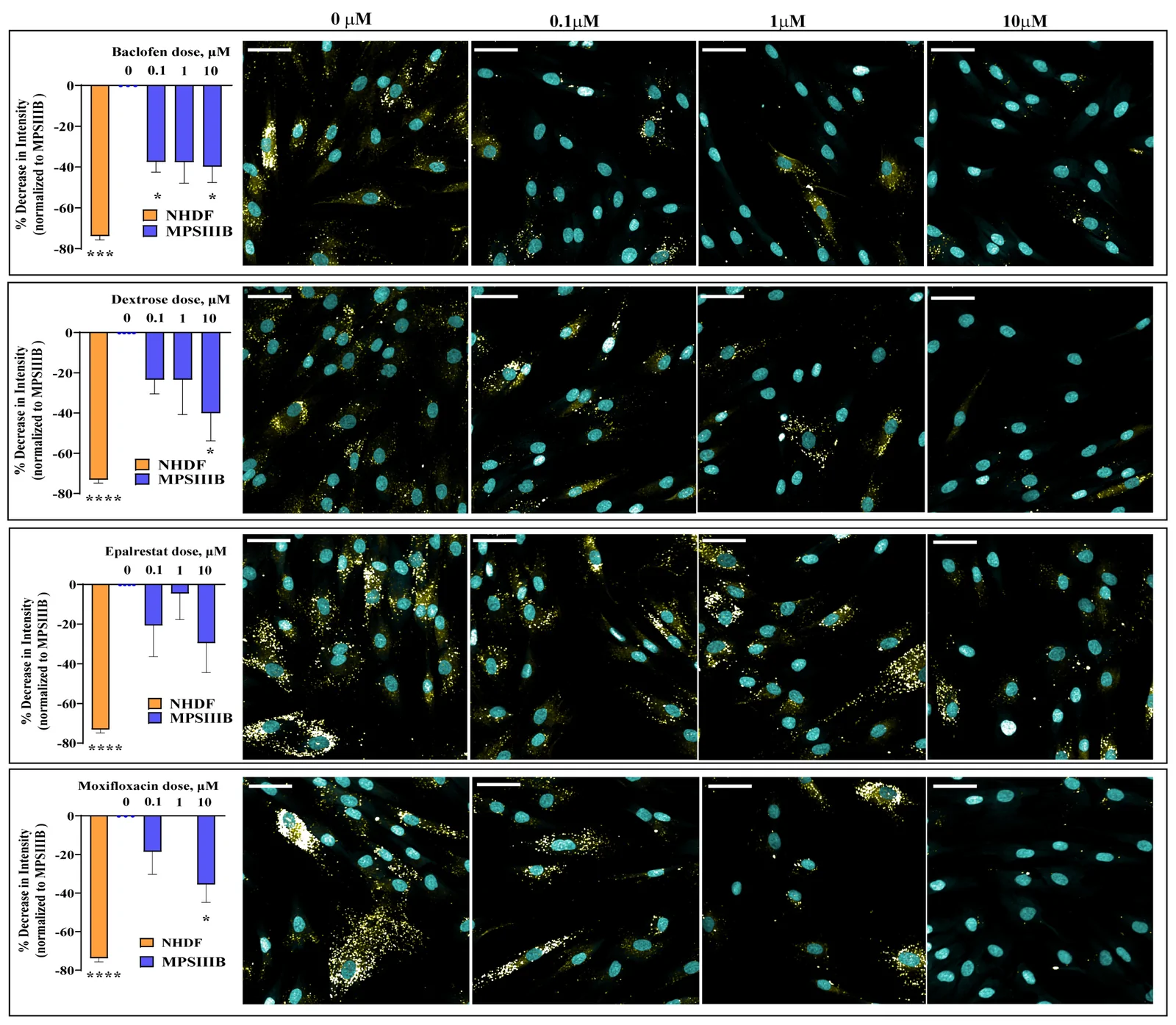
Dose plots for cherry-picked compounds in index patient fibroblasts. Left: Lysosomal staining intensity after 72 h of treatment with four compounds that met the initial screen criteria of at least 25% reduction in lysotracker-integrated staining intensity with less than 50% cell loss. Compounds were screened at 10 µM, 1 µM and 0.1 µM doses; *n* = 3 to 5 independent treatment wells that were averaged from 9 fields of view. Cyan, nuclei; Yellow, LysoTracker. The cytoplasmic channel was omitted to better visualize LysoTracker-positive puncta. Scale bar = 50 µm. Data are presented as mean +/− SEM. Right: Representative images for each treatment group. * *p* < 0.05, *** *p* < 0.001, **** *p* < 0.0001 by one-way ANOVA with Tukey’s multiple comparisons test.

Remarkably, four of these confirmed hits are orally available, clinically approved drugs: baclofen (a GABA-B receptor agonist), dextrose (a monosaccharide), epalrestat (an aldose reductase inhibitor), and moxifloxacin (an antibiotic). All four compounds produced a dose-dependent reduction in lysosomal accumulation in the index patient’s fibroblasts (Figure 4) while exhibiting minimal cytotoxicity (Figure S5A). The dose–response relationships were not strictly monotonic across all three concentrations tested; some compounds showed lower efficacy at 1 µM than at 0.1 µM, possibly reflecting non-linear target engagement, compound solubility at intermediate concentrations, or assay variability, and warrant further characterization. The remaining six confirmed hits were either not approved for therapeutic use or were unsuitable for systemic administration, though they may represent leads for future medicinal chemistry efforts (Figure S5B). To better understand the phenotypic effects of the confirmed hits, we analyzed individual LysoTracker-derived parameters, including spot count, spot area, and mean spot intensity, for each of the four clinically approved compounds identified in the primary screen (Figure S6). Across compounds, treatment predominantly reduced LysoTracker spot count and spot area, with minimal effects on mean spot intensity. This pattern suggests that compound activity was primarily associated with changes in lysosomal morphology and/or abundance rather than alterations in the fluorescence intensity of individual LysoTracker-positive compartments.

### 3.3. Confirmed Hits Demonstrate Profound Patient-Specific Efficacy

**Figure 5:**
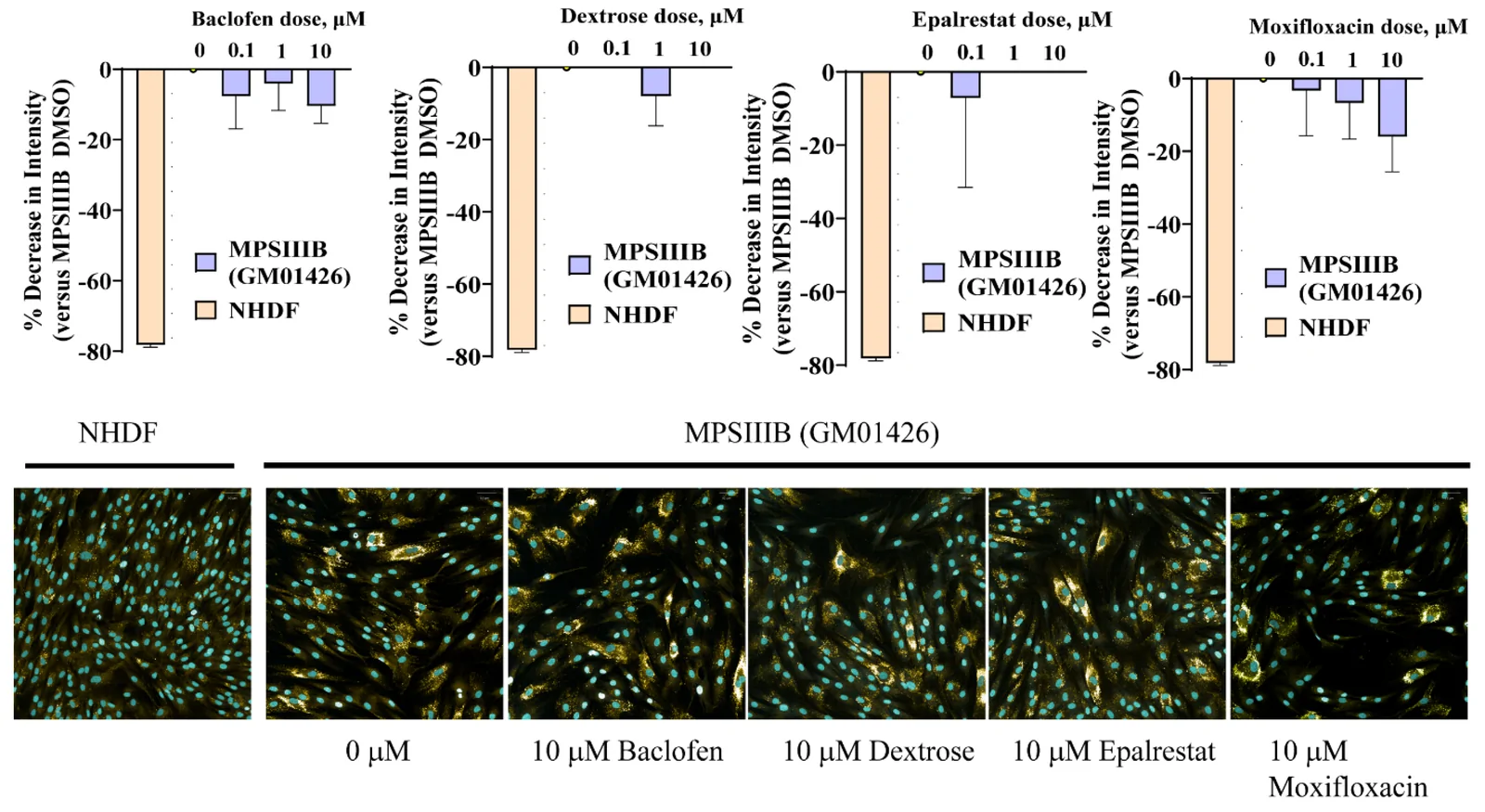
Dose plots for cherry-picked compounds in GM01426 fibroblasts. Lysosomal staining intensity after 72 h of treatment with cherry-picked compounds from index patient fibroblast screen. NHDF, MPSIIIB (GM01426), and MPSIIIB DMSO (0 µM) conditions are shown for reference. Compounds were screened at 10 µM, 1 µM and 0.1 µM doses; *n* = 4–7 independent treatment wells that were averaged from 9 fields of view. Data are presented as mean +/− SEM. Right: Representative images for each treatment group are shown below the graphs. Cyan, nuclei; Yellow, LysoTracker.

To determine if the identified hits represented a broadly applicable therapy for MPSIIIB, we tested all 10 confirmed compounds in fibroblasts from a second patient (GM01426) with a different NAGLU genotype. The results were striking. None of the four clinically approved drugs that were effective in the index patient’s cells—baclofen, dextrose, epalrestat and moxifloxacin—produced a significant reduction (>25%) in lysosomal accumulation in the second patient’s fibroblasts (Figure 5). These findings indicate a patient-specific therapeutic response. Of the six other compounds, only 6-chlorothymol showed greater than 25% reduction in LysoTracker staining at a 10µM dose, although this reduction did not achieve statistical significance by one-way ANOVA analysis. No reduction was seen with lower doses (Figure S5C). This divergence in drug responsiveness underscores the functional consequences of genetic heterogeneity on cellular phenotype and therapeutic vulnerability.

## 4. Discussion

Personalized drug screening in patient-derived dermal fibroblasts offers a pragmatic and rapid path to identifying potential therapeutic candidates for individuals with rare diseases who have no treatment options [19]. Patient fibroblasts offer key advantages for such screens: they are easy to culture and to scale up, and they retain the patient’s unique genetic as well as epigenetic signatures that may be lost in programmed cell models [20]. While screening in fibroblasts has limitations, including limited representation of disease-relevant cell types and tissue-specific context, it represents a powerful tool for initial discovery.

Efforts to find therapeutic options for MPSIIIB have been challenging, and treatment for this disease remains an unmet need. To our knowledge, no prior high-throughput screening (HTS) campaigns have been reported for MPSIIIB, likely due to technical challenges of quantifying its heterogeneous cellular phenotype. Quantifying measurable phenotypic differences between disease and non-disease cells can be challenging. Inherent differences within a cell, particularly when cell populations contain dominant and phenotypically distinct subpopulations, contribute to cell heterogeneity [21]. We observed that lysosomal accumulation in MPSIIIB cells appeared as large, variable clusters rather than discrete puncta, complicating simple counting-based analysis. We addressed this by developing a composite quantification algorithm integrating the area, number, and mean intensity of stained regions. This approach successfully captured the phenotype across the heterogeneous population, providing a sufficient signal window to screen focused compound libraries.

The primary therapeutic goal of this work was to identify drugs that correct the functional disease-state lysosomal phenotype in MPSIIIB patients, who by definition do not have functional NAGLU. In this context, the relevant therapeutic question is whether a compound restores the lysosomal phenotype in disease cells. As expected, we did not observe any decrease or change in LysoTracker staining intensity in NHDF cells upon treatment with δ-tocopherol (Figure 2) since healthy cells do not exhibit lysosomal expansion and there is no inflated signal for a compound to reduce. Therefore, the compound screen was performed in MPSIIIB patient cells. Direct biochemical HS quantification and NAGLU enzyme activity measurements in treated patient cells remain an important next step to confirm that the identified hits genuinely reduce substrate accumulation, and these are prioritized for follow-up studies.

Our screen identified ten confirmed hits from the NPC library, including four orally available clinically approved drugs: baclofen, dextrose, epalrestat, and moxifloxacin. While the mechanisms by which these compounds reduce LysoTracker staining in MPSIIIB cells remain to be fully established, their known pharmacology suggests plausible mechanistic hypotheses that could guide future investigation.

Baclofen is a selective GABA-B receptor agonist and CNS-penetrant drug already used to treat neurological conditions including spasticity in cerebral palsy [22,23,24]. Baclofen can partially cross the BBB, and oral administration at various doses produces therapeutic effects [25]. While intrathecal administration achieves efficacy at orders-of-magnitude lower concentrations than oral dosing, comparable therapeutic effects can be achieved by either route [26]. To our knowledge, a direct link between baclofen and lysosomal clearance has not been elucidated, but it is conceivable that baclofen may improve lysosomal burden indirectly. Alterations in cellular metabolism and inhibition of autophagy have been shown to rescue lysosomal pathology in MPSIIIB fibroblasts through activation of the PI3K/Akt signaling pathway [18]. Notably, baclofen has been reported to upregulate this pathway in a rat model of chronic cerebral hypoperfusion [27]. The same study further demonstrated that baclofen modulates autophagy and exerts neuroprotective effects [27]. The ability of baclofen to cross the BBB and its established neurological safety profile make it a particularly attractive candidate for further investigation in MPSIIIB, where both lysosomal and behavioral manifestations are prominent [25].

Dextrose is widely used clinically, administered orally or intravenously for glycemic control. While its use beyond glucose replacement is uncommon, there is emerging evidence for potential efficacy in treating chronic musculoskeletal conditions [28]. The mechanism by which dextrose might reduce lysosomal accumulation in MPSIIIB cells is not yet established; possible explanations include indirect modulation of the mTOR pathway through cellular glucose sensing or metabolic effects on lysosomal membrane homeostasis. This warrants further investigation before dextrose can be considered a confirmed therapeutic candidate.

Epalrestat is an aldose reductase inhibitor used primarily for treating diabetic peripheral neuropathy, approved in Japan, India, and China [29,30]. By reducing flux through the polyol pathway, epalrestat lowers sorbitol accumulation and associated oxidative stress [31]. Lysosomal function is highly sensitive to the cellular redox environment, and mitigation of oxidative burden has been proposed as a mechanism for secondary lysosomal improvement in metabolic storage disorders [32]. Notably, a recent high-throughput drug repurposing screen identified epalrestat as capable of increasing PMM2 enzyme activity in patient-derived fibroblasts and model organisms with PMM2-CDG [33], and a next-generation aldose reductase inhibitor subsequently demonstrated improved clinical outcomes in PMM2-CDG patients [34]. This precedent demonstrates that hits from repurposing screens—or their derivative compounds—can lead to clinically meaningful therapeutics for rare metabolic diseases, supporting the potential value of our finding.

Moxifloxacin is an FDA-approved fluoroquinolone antibiotic used for respiratory tract, skin, intra-abdominal, and ophthalmic infections. Fluoroquinolones have been reported to modulate autophagy and mitochondrial function, and some have shown activity in lysosomal disease models possibly through mitochondrial-lysosomal crosstalk or proteostasis effects. However, the FDA has issued safety warnings for moxifloxacin regarding potentially irreversible side effects including tendinitis, tendon rupture, and peripheral neuropathy [35], which must be carefully considered in any translational follow-up.

The six remaining hits—astromicin, denatonium benzoate, edrophonium chloride, ethoheptazine hydrochloride, 6-chlorothymol, and (2S,5R)-5-methyl-2-(propan-2-yl) cyclohexanone—are not currently in clinical use for therapeutic indications. Their relevance to MPSIIIB pathophysiology is not immediately apparent but their known mechanisms may provide insights into future medicinal chemistry efforts. Of particular interest, 6-chlorothymol is a phenolic compound with antiseptic properties and potential GABA-A modulating activity; preliminary studies in preclinical models suggest that it may function as an anticonvulsant [36], which is relevant given the neurological manifestations of MPSIIIB. It was the only compound to show any trend toward activity in both patient cell lines, suggesting it may act through a mechanism less dependent on the specific NAGLU genotype.

The most important finding of this study was the lack of efficacy of the identified compounds in a second MPSIIIB patient-derived fibroblast line. This result highlights the challenge that genetic heterogeneity poses for therapeutic development in rare diseases. The index patient carried a homozygous c.889C>T (p.Arg297Ter) nonsense mutation, whereas the second patient (GM01426) carried a homozygous c.457G>A (p.Glu153Lys) missense mutation. The two cell lines exhibited markedly different responses to the compounds identified in our screen. Any pharmacological effect observed in the index patient cells is unlikely to depend on restoration of substantial NAGLU activity and may instead involve mechanisms such as modulation of lysosomal biogenesis, autophagy flux, or downstream metabolic signaling pathways, consistent with the predominantly size- and area-based parameter changes observed following treatment (Figure S6). The divergent responses may reflect fundamental differences in the molecular consequences of these genotypes. The p.Arg297Ter variant is predicted to produce either a severely truncated protein or nonsense-mediated mRNA decay, resulting in little or no functional enzyme. In contrast, the p.Glu153Lys missense variant may impair protein folding or stability while retaining partial NAGLU activity. If the compound activity depended primarily on suppression of premature translation termination, some degree of efficacy might be expected in both cell lines. This possibility is relevant because nonsense mutation suppression has previously been shown to restore partial enzyme activity and reduce GAG accumulation in lysosomal storage disease models [37,38]. However, the absence of activity in GM01426 suggests that additional factors may influence therapeutic responsiveness. Several non-mutually exclusive mechanisms could account for this divergence: (1) the residual NAGLU activity already present in GM01426 may alter the cellular response threshold such that a modest additional rescue is insufficient to shift the measurable LysoTracker phenotype; (2) the p.Glu153Lys missense mutation may modify the protein folding or cellular stress environment in a way that changes drug sensitivity; (3) secondary cellular adaptations to partial versus complete enzyme loss differ substantially and may fundamentally alter the lysosomal stress response and pharmacological vulnerability; and (4) epigenetic differences between cell lines may influence transcriptional responses to treatment independently of genotype. Additional studies will be required to distinguish among these possibilities and define the mechanisms underlying the observed genotype-dependent effects.

More broadly, these findings support the concept that therapeutic response in MPSIIIB may depend on the underlying mutation and cellular context rather than disease diagnosis alone. A similar principle has been established in cystic fibrosis, where therapeutic efficacy is determined by the specific molecular defect affecting CFTR function. For example, potentiators such as ivacaftor are effective for gating mutations, whereas processing mutations require correctors in combination with potentiation [39]. While the mechanisms are distinct, this precedent illustrates how genotype can be a critical determinant of treatment responsiveness in monogenic diseases. Our results therefore support the use of genotype-stratified patient-derived screening approaches and highlight the potential value of N-of-1 drug repurposing strategies for genetically heterogeneous disorders such as MPSIIIB.

Despite this challenge of patient-specific responses, personalized drug repurposing screens may represent the most cost-effective and time-efficient strategy for identifying therapeutics for rare disease patients, particularly when conventional drug development pathways have failed. The successful development of individualized oligonucleotide therapy for a single patient with Batten disease, which received FDA approval under the expanded access pathway [40], demonstrates the feasibility of this approach. As technologies for rapid screening and antisense oligonucleotide synthesis continue to advance, personalized therapeutic strategies may become increasingly practical for rare disease patients.

Our study has several limitations that should be acknowledged. First, because screening was performed only in fibroblasts from the index patient, our findings on specific drugs are not generalizable to all MPSIIIB patients. Second, the LysoTracker staining assay is an indirect readout of lysosomal dysfunction driven by GAG accumulation, and a reduction in signal may reflect mechanisms other than direct HS clearance. Although LysoTracker staining provides a useful phenotypic readout of lysosomal abnormalities in MPSIIIB cells, it does not specifically assess HS endocytosis or distinguish intracellular HS from extracellular or membrane-associated pools. Antibody-based HS immunofluorescence did not yield quantifiable differences between NHDF and MPSIIIB cells in our hands (Figure S4), consistent with the known technical limitations of this approach and underscoring the need for direct biochemical HS quantification and compartment-specific HS imaging in future studies. Third, the mechanisms of action for our hits remain to be established experimentally, and the mechanistic hypotheses proposed here will require dedicated follow-up studies. Analysis of individual LysoTracker parameters showed that the confirmed hit compounds predominantly reduced spot count and spot area, with minimal effects on mean spot intensity. This pattern suggests that the observed phenotypic effects were driven primarily by changes in the number and/or size of LysoTracker-positive compartments rather than by changes in fluorescence intensity per compartment; however, the underlying biological mechanisms remain to be determined. Reduced HS uptake, altered endolysosomal trafficking, changes in lysosomal turnover or biogenesis, altered lysosomal function, or other related mechanisms could all contribute to a reduction in lysosomal burden. Direct biochemical quantification of HS and measurement of NAGLU enzyme activity in treated cells will therefore be essential to determine whether the observed phenotypic rescue reflects on-target modulation of the underlying disease pathology. Fourth, although the fibroblast model is valuable for initial discovery, it has inherent limitations in representing disease-relevant neuronal cell types and tissue-specific context. Validation in iPSC-derived neurons or relevant animal models will therefore be an important future direction.

In conclusion, we successfully implemented a personalized drug repurposing screen for an MPSIIIB patient, identifying four clinically approved compounds that improve the patient’s disease-state cellular phenotype as measured by an indirect lysosomal accumulation assay. The patient-specific nature of these compounds highlights the profound challenges posed by genetic heterogeneity and suggests that mutation-specific factors may be important determinants of drug response in MPSIIIB. Our work demonstrates the feasibility of rapid, N-of-1 screening for rare diseases and supports a precision medicine framework in which therapeutic discovery is guided by patient-specific genetic and cellular context, with the goal of identifying individualized treatment strategies for patients who currently have limited therapeutic options.

## Figures and Tables

**Table 1:** Confirmed Hits.

Structure	Name and ID	Known Mechanism	Clinical Status
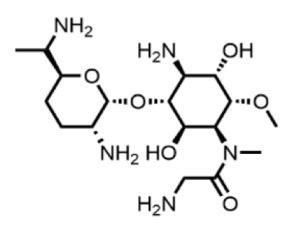	Astromicin NCGC00185750	Aminoglycoside antibiotic -binds the bacterial 30S ribosomal subunit, disrupting protein synthesis	Approved in Japan for gram- negative bacterial infections. Not widely used
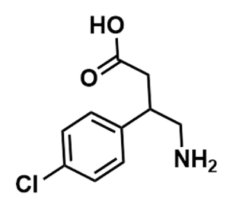	Baclofen NCGC00015156	Selective GABA-B receptor agonist	Approved worldwide for spasticity. Used orally and intrathecally
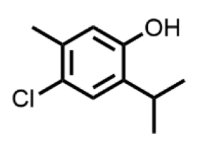	6-Chlorothymol NCGC00159384	A phenolic antiseptic that disrupts microbial cell membranes and proteins	Used in formulations for topical disinfectants and antiseptic
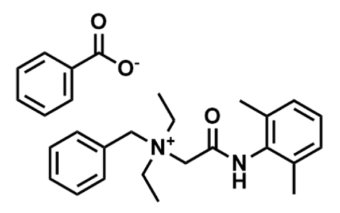	Denatonium benzoate NCGC00091886	Agonist of human bitter taste receptors	Used as a bitter additive in clinical trials in placebos to match the bitter taste of certain medications
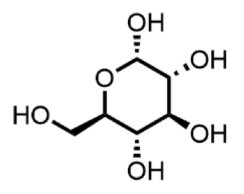	Dextrose NCGC00160621	Glucose monosaccharide used as metabolic substrate	Widely approved and used in intravenous solutions for carbohydrate and fluid provision
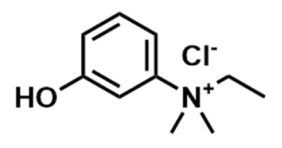	Edrophonium Chloride NCGC00015409	Short-acting acetylcholinesterase inhibitor	Historically used for the diagnosis for myasthenia gravis. Rarely used in current practice
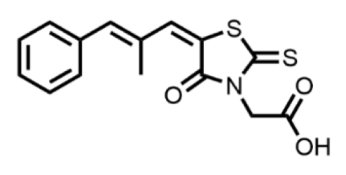	Epalrestat NCGC00164613	Aldose reductase inhibitor	Approved in Japan, China and India for diabetic neuropathy
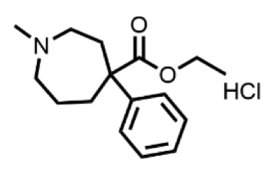	Ethoheptazine hydrochloride NCGC00183870	Opioid receptor agonist analgesic	Treatment for headache or musculoskeletal disorders. Discontinued in USA
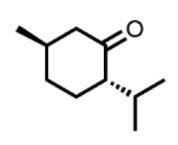	(2S,5R)-5-Methyl -2-(propan-2-yl) cyclohexanone (Menthone) NCGC0009560	Not well established	Not an approved therapeutic drug. A naturally occurring compound with anti-inflammatory, antimicrobial and possibly neuroprotection properties
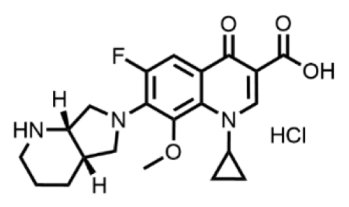	Moxifloxacin (Hydrochloride) NCGC00271749	Blocks bacterial replication—DNA topoisomerase II and IV Inhibitor	A synthetic fluoroquinolone used to treat bacterial infections (respiratory tract, skin, intra- abdominal and ophthalmic). Approved but with safety warnings

## Data Availability

Data from the screen have been deposited in PubChem and are publicly available at (https://pubchem.ncbi.nlm.nih.gov/bioassay/2202682, Accessed on 11 May 2026).

## Supplementary Materials

The following supporting information can be downloaded at: https://www.mdpi.com/article/10.3390/jpm16070369/s1, Figure S1: 1536-well plate PDL vs. non-coated plates; Figure S2: Individual parameters used to calculate integrated lysosomal intensity; Figure S3: Assays to determine differences between NHDF and MPSIII fibroblasts; Figure S4: Heparan sulfate immunostaining in NHDF and MPSIIIB fibroblasts; Figure S5: Assay hits in index patient and GM0146 MPSIIIB fibroblasts; Figure S6: Individual LysoTracker parameters following treatment with cherry-picked compounds.
