# Correction: McDaniel et al. Personalized Drug Repurposing Screen Identifies Patient-Specific Therapeutic Candidates for Mucopolysaccharidosis Type IIIB. *J. Pers. Med.* 2026, *16*, 369

**DOI:** 10.3390/jpm16100528

**Published:** 2026-10-13

**Authors:** Kathleen D. McDaniel, Neda Ghousifam, Rodney A. Bowling, Catherine Z. Chen, Wei Zheng, Zeenat A. Shyr

**Affiliations:** 1 National Center for Advancing Translational Sciences, National Institutes of Health, 9800 Medical Center Drive, Rockville, MD 20850, USAcatherine.chen@nih.gov (C.Z.C.); 2 AlphaRose RareLabs, 1401 Lavaca St, Austin, TX 78701, USA; ghosifam@gmail.com (N.G.); rodney@alpharose.com (R.A.B.J.)

## 1. Text Correction

There was an error in the original publication [1]. The second MPSIIIB patient fibroblast line, GM01426, was erroneously described as a compound heterozygous genotype. The correct genotype is homozygous for a G>A substitution at nucleotide 457 of the NAGLU gene (457G>A), resulting in the replacement of glutamic acid with lysine at codon 153 [p.Glu153Lys (E153K)].

Corrections have been made.

Abstract, Sentence 5

Hits that reduced lysosomal accumulation by at least 25% with minimal cytotoxicity were validated and subsequently tested for efficacy in fibroblasts from a second patient with a different NAGLU genotype.

2.1. Patient Information, Paragraph 1, Sentences 4–6

A second MPSIIIB patient fibroblast line, GM01426, was purchased from the Coriell Institute for Medical Research (Camden, NJ, USA). The GM01426 fibroblast line has reduced α-N-acetylglucosaminidase activity and is homozygous for the NAGLU c.457G>A variant, resulting in the p.Glu153Lys (E153K) substitution [13]. This variant has been reported in ClinVar as likely pathogenic or pathogenic (Variation ID: 1067431; VCV001067431.11).

3.3. Confirmed Hits Demonstrate Profound Patient-Specific Efficacy, Paragraph 1, Sentence 1

To determine if the identified hits represented a broadly applicable therapy for MPSIIIB, we tested all 10 confirmed compounds in fibroblasts from a second patient (GM01426) with a different NAGLU genotype.

4. Discussion, Paragraph 10

The most important finding of this study was the lack of efficacy of the identified compounds in a second MPSIIIB patient-derived fibroblast line. This result highlights the challenge that genetic heterogeneity poses for therapeutic development in rare diseases. The index patient carried a homozygous c.889C>T (p.Arg297Ter) nonsense mutation, whereas the second patient (GM01426) carried a homozygous c.457G>A (p.Glu153Lys) missense mutation. The two cell lines exhibited markedly different responses to the compounds identified in our screen. Any pharmacological effect observed in the index patient cells is unlikely to depend on restoration of substantial NAGLU activity and may instead involve mechanisms such as modulation of lysosomal biogenesis, autophagy flux, or downstream metabolic signaling pathways, consistent with the predominantly size- and area-based parameter changes observed following treatment (Figure S6). The divergent responses may reflect fundamental differences in the molecular consequences of these genotypes. The p.Arg297Ter variant is predicted to produce either a severely truncated protein or nonsense-mediated mRNA decay, resulting in little or no functional enzyme. In contrast, the p.Glu153Lys missense variant may impair protein folding or stability while retaining partial NAGLU activity. If the compound activity depended primarily on suppression of premature translation termination, some degree of efficacy might be expected in both cell lines. This possibility is relevant because nonsense mutation suppression has previously been shown to restore partial enzyme activity and reduce GAG accumulation in lysosomal storage disease models [37,38]. However, the absence of activity in GM01426 suggests that additional factors may influence therapeutic responsiveness. Several non-mutually exclusive mechanisms could account for this divergence: (1) the residual NAGLU activity already present in GM01426 may alter the cellular response threshold such that a modest additional rescue is insufficient to shift the measurable LysoTracker phenotype; (2) the p.Glu153Lys missense mutation may modify the protein folding or cellular stress environment in a way that changes drug sensitivity; (3) secondary cellular adaptations to partial versus complete enzyme loss differ substantially and may fundamentally alter the lysosomal stress response and pharmacological vulnerability; and (4) epigenetic differences between cell lines may influence transcriptional responses to treatment independently of genotype. Additional studies will be required to distinguish among these possibilities and define the mechanisms underlying the observed genotype-dependent effects.

## 2. Reference Correction

The original reference 13 has been changed to the following:

13. Huang, W.; Xu, M.; Li, R.; Baskfield, A.; Kouznetsova, J.; Beers, J.; Zou, J.; Liu, C.; Zheng, W. An induced pluripotent stem cell line (TRNDi006-A) from a MPS IIIB patient carrying homozygous mutation of p.Glu153Lys in the NAGLU gene. *Stem Cell Res.* **2019**, *37*, 101427.

The authors state that the scientific conclusions are unaffected. This correction was approved by the Academic Editor. The original publication has also been updated.
