# Supplementary material for: Personalized Drug Repurposing Screen Identifies Patient-Specific Therapeutic Candidates for Mucopolysaccharidosis Type IIIB
Source: J Pers Med. 2026 Jul 8;16(7):369. doi: 10.3390/jpm16070369 (PMC13412476; doi:10.3390/jpm16070369)
Supplement: Supplementary file 1 [file jpm-16-00369-s001.zip › Figure legends-supplementary final.pdf]

Figure legends- supplementary

Figure S1. 1536-well plate PDL vs non-coated plates
Top: Representative images for NHDF and MPSIIIB fibroblasts in 1536-well plates with or without PDL coating. Blue, nuclei; Magenta, high content cell mask; Yellow, lysotracker. Bottom: quantification of nuclei counts 72 hours after plating 200 cells; n=255 independent wells.

Figure S2. Individual parameters used to calculate integrated lysosomal intensity
Differences between NHDF and MPSIIIB for lysosomal spot number, area, and intensity are shown in separate graphs for each cell density tested. ****P<0.0001 by Students *t*-test

Figure S3. Assays to determine differences between NHDF and MPSIII fibroblasts
(A) Nile Red staining. Top: Representative images showing Nile Red fluorescence at an excitation emission wavelength of 450/528 nm to detect neutral intracellular lipids. Bottom: Merged with fluorescence detected at an excitation emission wavelength of 550/640 nm. Blue, nuclei; Green, Nile red (non-polar lipids); Pink, Nile red (polar lipids). Below: quantification of fluorescence staining intensity at ex/em 450/528; n=32 independent wells. (B) Average well fluorescence reading at 568 nm wavelength using Clariostar plate reader. Each data point represents an independent well; n=128 independent wells. ****P<0.0001 by Students *t*-test

Figure S4. Heparan sulfate immunostaining in NHDF and MPSIIIB fibroblasts
Representative images showing heparan sulfate immunostaining. Blue, nuclei; Green, Heparan sulfate; Red, CellMask red.

Figure S5. Assay hits in index patient and GM0146 MPSIIIB fibroblasts.
(A) Cell viability (n=4). (B) lysosomal staining intensity in TCAR fibroblasts. (C) Lysosomal staining intensity in GM01426 fibroblasts n=4-7 in each. *P<0.05; **P< 0.01; ***P<0.001; ****P< 0.0001 by one-way ANOVA with Tukey's multiple comparisons test.

Figure S6. Individual LysoTracker parameters following treatment with cherry-picked compounds
Reduction in Spot count, Spot area, and Mean Spot Intensity for each of the four confirmed clinically approved drugs; n=4-7 in each. *P< 0.05; **P < 0.01; ***P < 0.001 by one-way ANOVA.
