# Supplementary figures and images for: Personalized Drug Repurposing Screen Identifies Patient-Specific Therapeutic Candidates for Mucopolysaccharidosis Type IIIB
Source: J Pers Med. 2026 Jul 8;16(7):369. doi: 10.3390/jpm16070369 (PMC13412476; doi:10.3390/jpm16070369)

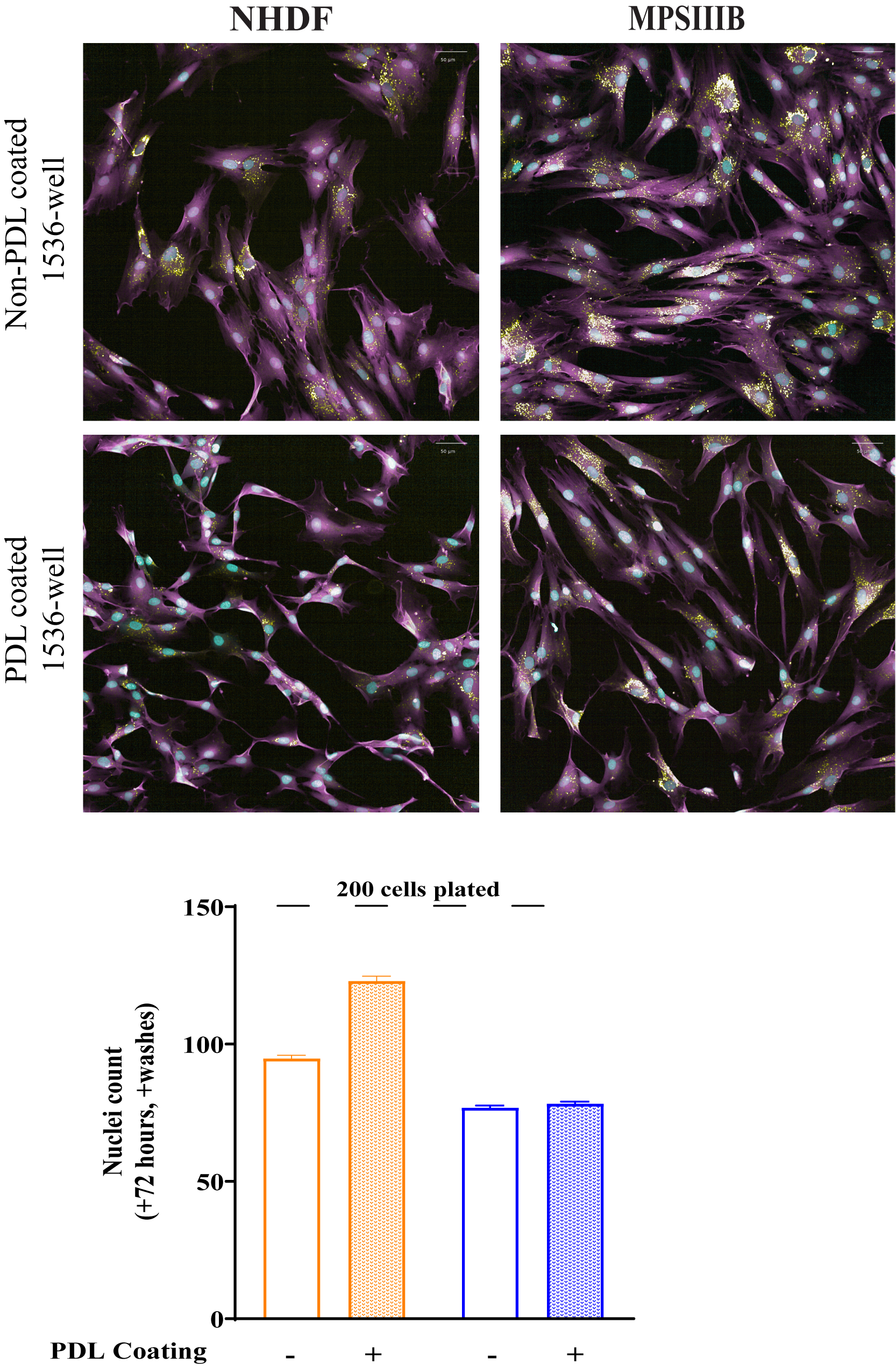

Supplement: Supplementary file 1 [file jpm-16-00369-s001.zip › Figure S1- 20260622.tif]

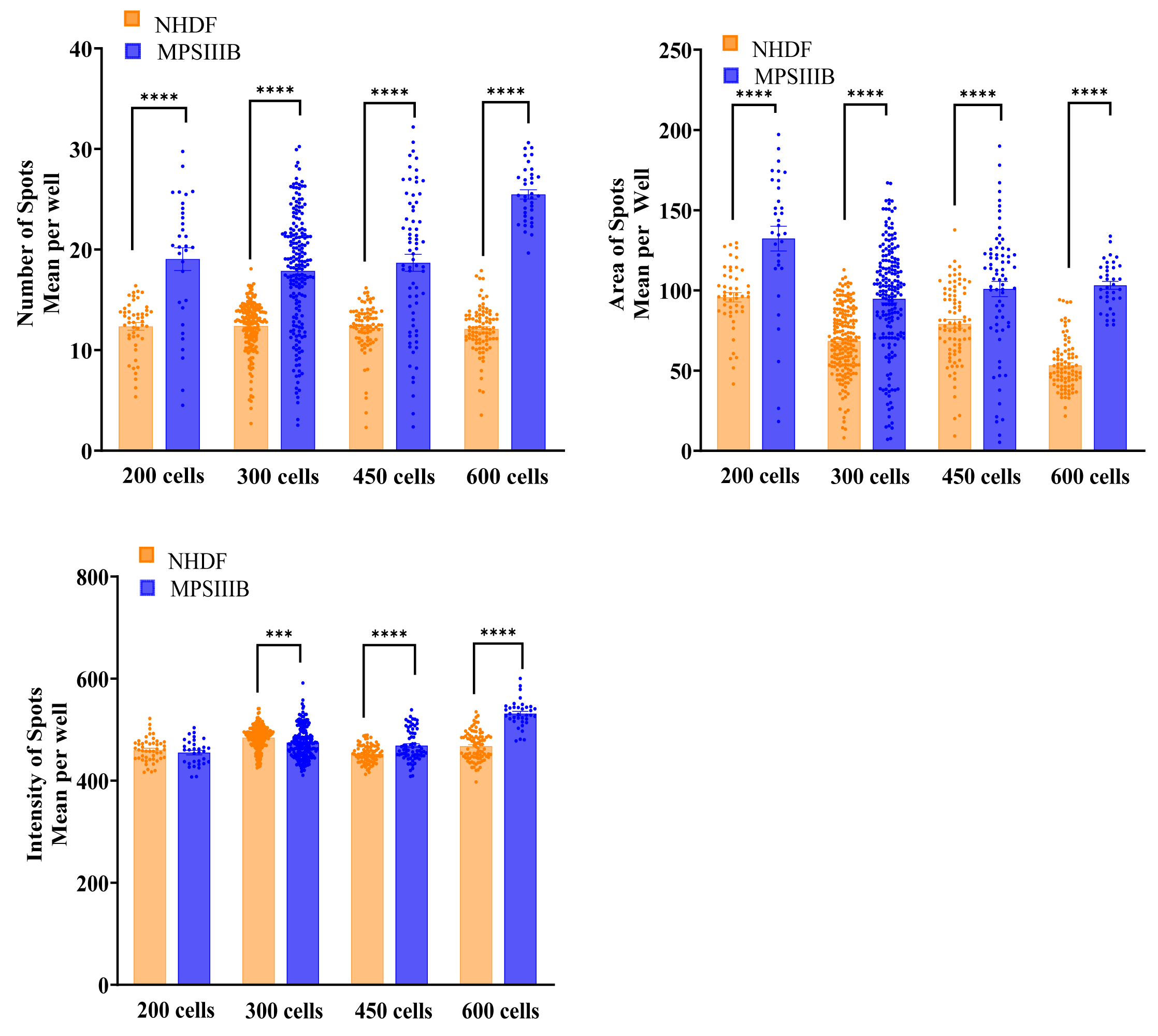

Supplement: Supplementary file 1 [file jpm-16-00369-s001.zip › Figure S2.tif]

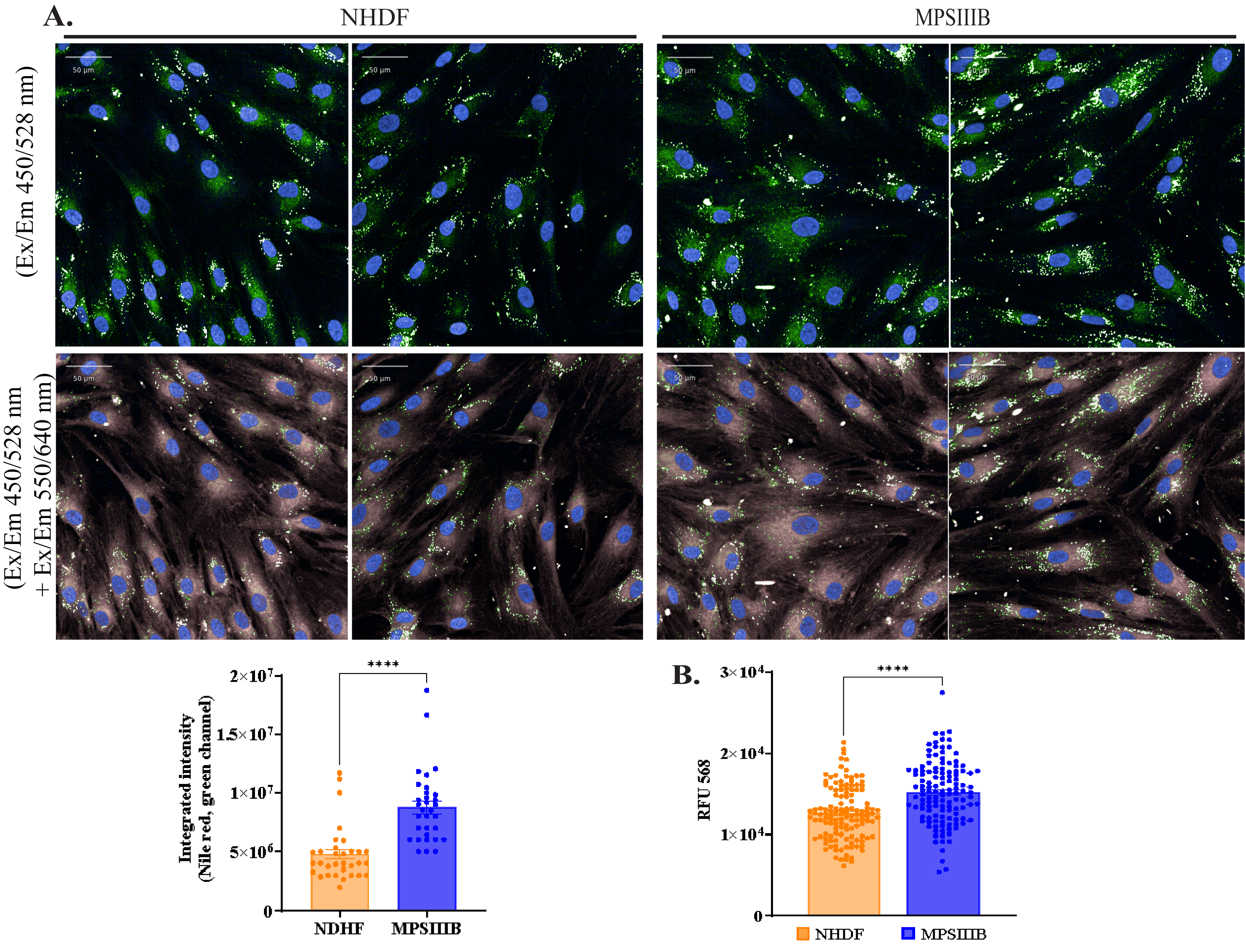

Supplement: Supplementary file 1 [file jpm-16-00369-s001.zip › Figure S3- updated 20260623.tif]

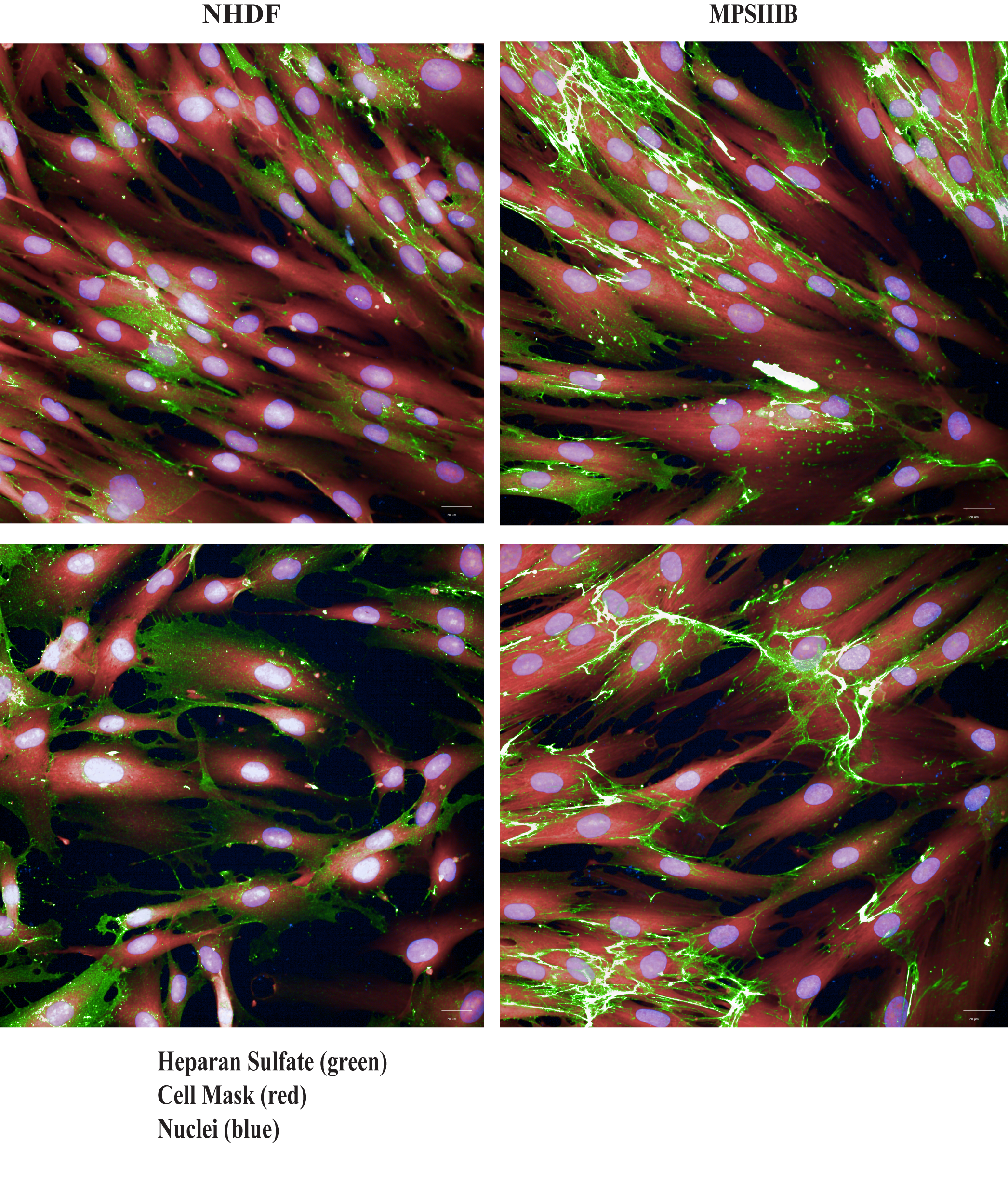

Supplement: Supplementary file 1 [file jpm-16-00369-s001.zip › Figure S4.tif]

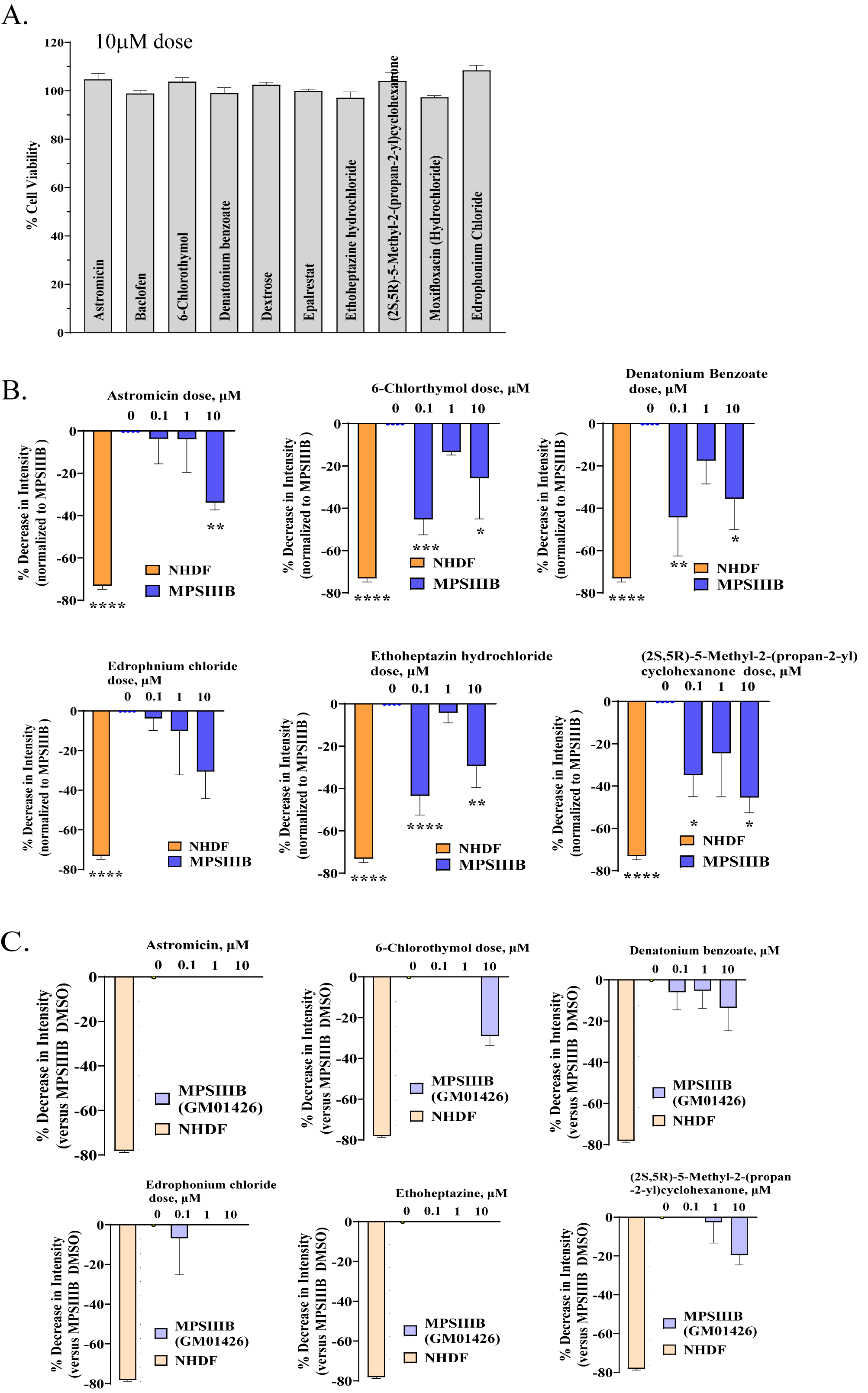

Supplement: Supplementary file 1 [file jpm-16-00369-s001.zip › Figure S5.tif]

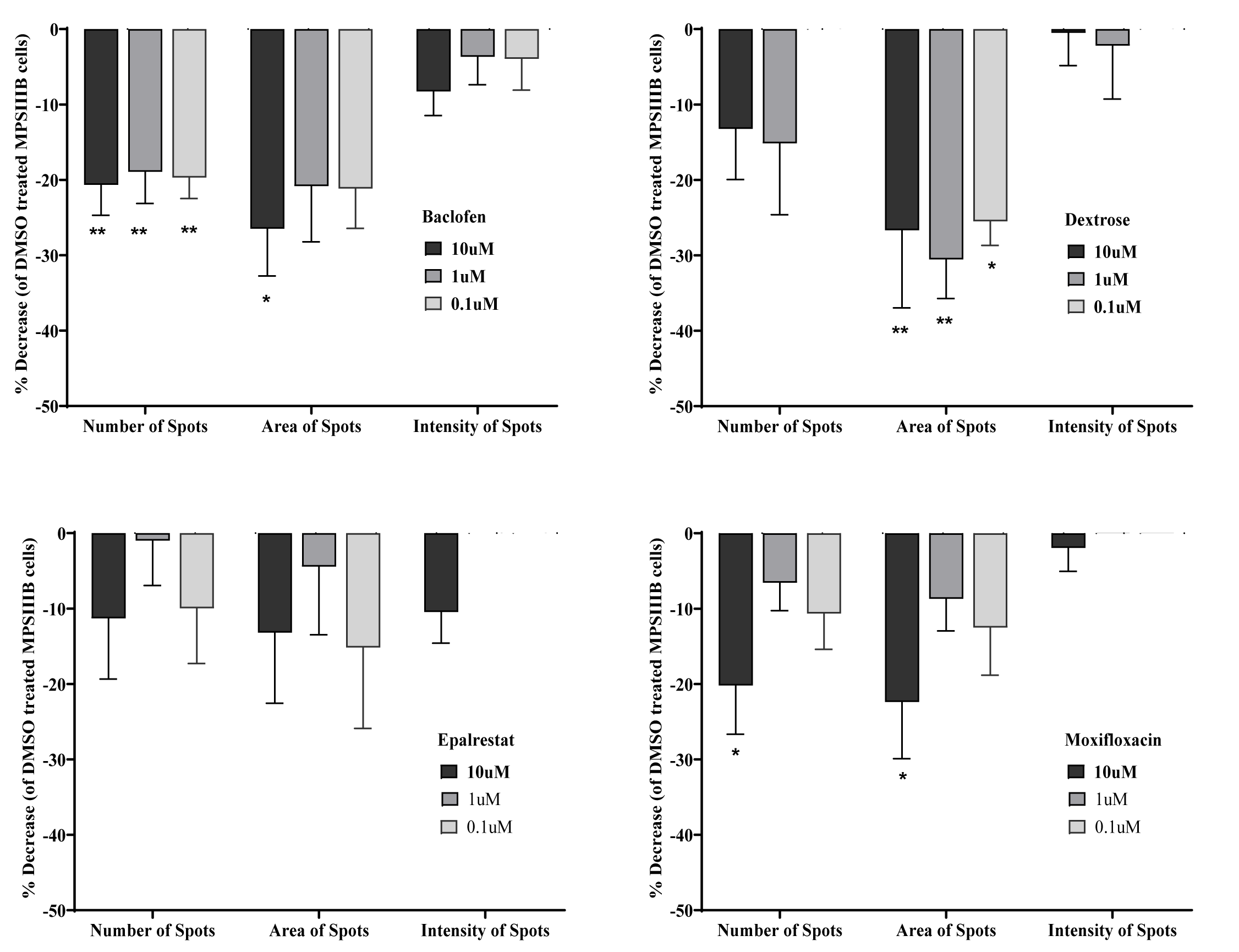

Supplement: Supplementary file 1 [file jpm-16-00369-s001.zip › Figure S6.tif]
